# Lipopolysaccharide (LPS) stimulation of Pancreatic Ductal Adenocarcinoma (PDAC) and macrophages activates the NLRP3 inflammasome that influences the levels of pro-inflammatory cytokines in a co-culture model

**DOI:** 10.1080/15384047.2023.2284857

**Published:** 2023-11-29

**Authors:** Hariss G Paremes Sivam, Beek Yoke Chin, Sook Yee Gan, Jia Hao Ng, Agnes Gwenhure, Elaine Wan Ling Chan

**Affiliations:** a School of Health Sciences, International Medical University, Kuala Lumpur, Malaysia; b Institute for Research, Development and Innovation, International Medical University, Kuala Lumpur, Malaysia; c Department of Life Science, School of Pharmacy, International Medical University, Kuala Lumpur, Malaysia; d School of Postgraduate Studies, International Medical University, Kuala Lumpur, Malaysia

**Keywords:** Pancreatic ductal adenocarcinoma, tumor associated macrophages, LPS-induced inflammation, NLRP3 inflammasome, NLRP3 inflammasome inhibitor, MCC950

## Abstract

Modified macrophages, tumor-associated macrophages (TAMs), are key contributors to the survival, growth, and metastatic behavior of pancreatic ductal adenocarcinoma (PDAC) cells. Central to the role of inflammation and TAMs lies the NLRP3 inflammasome. This study investigated the effects of LPS-stimulated inflammation on cell proliferation, levels of pro-inflammatory cytokines, and the NLRP3 inflammasome pathway in a co-culture model using PDAC cells and macrophages in the presence or absence of MCC950, a NLRP3-specific inhibitor. The effects of LPS-stimulated inflammation were tested on two PDAC cell lines (Panc 10.05 and SW 1990) co-cultured with RAW 264.7 macrophages. Cell proliferation was determined using the MTT assay. Levels of pro-inflammatory cytokines, IL-1β, and TNF-α were determined by ELISA. Western blot analyses were used to examine the expression of NLRP3 in both PDAC cells and macrophages. The co-culture and interaction between PDAC cell lines and macrophages led to pro-inflammatory microenvironment under LPS stimulation as evidenced by high levels of secreted IL-1β and TNF-α. Inhibition of the NLRP3 inflammasome by MCC950 counteracted the effects of LPS stimulation on the regulation of the NLRP3 inflammasome and pro-inflammatory cytokines in PDAC and macrophages. However, MCC950 differentially modified the viability of the metastatic vs primary PDAC cell lines. LPS stimulation increased PDAC cell viability by regulating the NLRP3 inflammasome and pro-inflammatory cytokines in the tumor microenvironment of PDAC cells/macrophages co-cultures. The specific inhibition of the NLRP inflammasome by MCC950 effectively counteracted the LPS-stimulated inflammation.

## Introduction

Pancreatic Ductal Adenocarcinoma (PDAC) is becoming a growing concern on two key fronts: its high incidence-to-mortality ratio of 94% and its insidious onset of associated gastrointestinal, constitutional, and thrombotic symptoms, all of which have had a negative impact on the quality of life in affected patients.^1^ Most cases of PDAC are sporadic in nature. Each of the exogenous risk factors can be associated with acinar injury and chronic inflammation.^2,3^ Repeated episodes of injury are, therefore, associated with cycles of inflammation, which in turn lead to metaplasia and genomic instability, particularly resulting in oncogenic activation of KRAS.^4–6^ KRAS activation leads to the establishment of pancreatic intraepithelial neoplasia (PanIN)^7^ and maintains the constitutive activation of NF-κB, the master inflammatory transcription factor. NF-κB regulates the expression of inflammasomes, pro-inflammatory cytokines, and anti-apoptotic factors and enhances mitogenic EGF receptor signaling in transformed cells.^5,8–12^ This persistent inflammatory environment can facilitate the loss of tumor suppressors, leading to progression from PanIN to PDAC.^13^

Indeed, a conducive microenvironment is important to facilitate the survival of tumor cells, and co-existing along with tumor cells are macrophages of a specific phenotype termed tumor-associated macrophages (TAMs). Studies have established that the detection of TAMs is correlated with the malignant phenotype of PDAC.^14^ Interestingly, the phenotype of TAM populations in the tumor microenvironment varies according to the stage of the tumor and the dynamic biophysical features within micro-niches of the tumor, suggesting that crosstalk between tumor cells and TAMs does exist.^15^ In fact, exosomes derived from PDAC cells in hypoxic niches induced the M2 polarization of TAMs, which could be a pro-tumor phenotype because they facilitated tumor progression by promoting angiogenesis, desmoplasia, and metastasis and suppressing the anti-tumorigenic immune response in the tumor microenvironment, as well as regulated epithelial-to-mesenchymal transition (EMT) in pancreatic cancer cell migration and metastasis.^16–18^

The central mechanism driving inflammation is proposed to be orchestrated by the NLR pyrin domain-containing 3 (NLRP3) inflammasome, which is made up of the NLRP3 protein, an apoptosis-associated speck-like protein containing a caspase recruitment domain (ASC) and procaspase-1.^19^ NLRP3 inflammasome is markedly upregulated in pancreatic cancer.^20^ Pattern recognition receptors (PRRs) detect damage-associated molecular patterns (DAMPS) and lipopolysaccharide (LPS), leading to the activation of NF-κB with subsequent upregulation of NLRP3 inflammasome.^21,22^ The activation of the NLRP3 inflammasome requires a second signal provided by a wide variety of cellular or molecular stimuli, such as adenosine triphosphate (ATP).^23^ Activation of the inflammasome leads to the cleavage of precursors pro-IL-1β and pro-IL-18 into their active forms, i.e., IL-1β and IL-18, which have multiple roles in tumors.^24–26^

The NLRP3 inflammasome has been documented to play an important role in PDAC and is found to be markedly upregulated in macrophages in the PDAC microenvironment. It has been postulated that NLRP3 signaling within tumor-associated macrophages (TAMs) drives the polarization of CD4^+^ T cells in the tumor microenvironment toward an immune-suppressive phenotype. Blockade of NLRP3 signaling in TAMs increased the CD8/CD4 T cell ratio, and the CD4^+^ T cells themselves reprogrammed to the T_h_1 phenotype instead of the Treg phenotype commonly found in PDAC microenvironment, thereby enhancing anti-tumor immunity.^20^ Also, the NLRP3 inflammasome signaling in TAMs downregulates the expression of PD-1 receptors on T cells by inducing T cell anergy due to exhaustive induction of the PD-1 receptors. Interruption of NLRP3 signaling upregulates PD-1 receptors on T cells again, indicating restored sensitivity to tumor antigens and to PD-1 inhibition chemotherapy.^20,27^ These effects of NLRP3 inflammasome in PDAC are an addition to its role in producing IL-1β, which in turn activates NF- κB and STAT3, which have been shown to upregulate pro-survival factors.^8,28,29^ The role of IL-1β has been well documented in pancreatic cancer and is confirmed to be elevated even in precursor lesions such as pancreatic cysts.^30^ IL-1β promotes M2-like tumor-associated macrophages (TAM) accumulation, desmoplasia and suppresses anti-tumor immunity.^31^ Blockade of IL-1β reverses the M2-like phenotype polarization of TAMs, and in favorable scenarios, the repolarized TAMs with active NLRP3 inflammasomes and prior exposure to PDAC antigens present these PDAC antigens to T cells, enhancing anti-tumor immunity.^20^ The increase in local levels of IL-1β regulates the secretory activities of PSC-derived inflammatory cancer-associated fibroblasts (iCAFs).^20^ iCAFs secrete chemokines such as CCL2 that attract monocytes and polarize them toward the M2 TAM phenotype. In addition to this, iCAFs produce IL-6 and CXCL12, which suppress intra-tumoral immunity and CD8^+^ T cell immunity.^31,32^

Tumor necrosis factor-alpha (TNF-α) is an integral component of the innate immune system. Despite being initially studied as an anti-tumor cytokine, in recent times, chronic production of TNF-α in low concentrations has been shown to promote tumor survival, proliferation, invasion, and metastasis through various signaling pathways.^33,34^ Conceptually, TNF-α is postulated to be an important player in inflammation-induced carcinogenesis.^35,36^ Studies have documented the ability of TNF-α to regulate NLRP3 inflammasome expression and synergize with IL-1β, effectively sustaining a pro-inflammatory microenvironment.^37,38^ Hence, it merits further analysis and discussion to better understand the role of TNF-α in the tumor microenvironment simulated by the co-culture between PDAC cells and macrophages.

Interestingly, MCC950 could inhibit ATP hydrolysis by the NACHT (NAIP, C2TA, HET and TP1) domain in the NLRP3 protein, forcing the NLRP3 protein into a ‘closed’ conformer, inhibiting its oligomerization with ASC, and ultimately affecting NLRP3 inflammasome assembly and activation.^39^ The therapeutic potential of MCC950 in inflammation-related cancers has been highlighted.^40,41^ In addition, NLRP3 blockade in TAMs led to an increase in local CD8^+^/CD4^+^ T cell ratio, and the CD4^+^ T cells themselves reprogrammed to the T_h_1 phenotype instead of the suppressive Treg phenotype commonly found in PDAC microenvironment, thereby enhancing anti-tumor immunity.^20^

Thus, this study aimed to study the inhibitory effects of MCC950 on the activation of NLRP3 inflammasome in LPS-stimulated PDAC and TAM cells under co-culture conditions. The effects of the interaction between PDAC and TAM cells on the activation of NLRP3 inflammasome, production of IL-1β and TNF-α and PDAC cell proliferation were investigated. This study, when coupled with further studies in the future, will enable us to better understand the regulation of the tumor microenvironment between PDAC cells and TAM.

## Methods

### Preparation of lipopolysaccharide (LPS), adenosine triphosphate (ATP), and NLRP3-specific inhibitors (MCC950)

Lipopolysaccharide and adenosine triphosphate (ATP), purchased from ThermoFisher, USA, were diluted with phosphate-buffered saline (PBS) to a stock solution of 1 mg/mL and 50 µM, respectively.

MCC950 (Sigma, USA) was initially dissolved in dimethyl sulfoxide (DMSO; Calbiochem, USA) to produce a stock solution of 1000 µM followed by dilution with Roswell Park Memorial Institute Medium (RPMI 1640; Gibco, USA) serum-free media (SFM) to a concentration of 100 μM.

### Treatments of PDAC cells co-cultured with RAW 264.7 macrophages

RAW 264.7 murine macrophages (ATCC® TIB-71™) and two PDAC cell lines, namely primary Panc 10.05 PDAC cells and metastatic SW 1990 PDAC cells, were acquired from the American Type Culture Collection (ATCC). All cell lines were maintained in 5% CO_2_ at 37°C using a complete culture medium consisting of RPMI with 10% heat-inactivated fetal bovine serum (FBS; (ATCC), VA) and 1% penicillin-streptomycin (Lonza, USA).

To investigate the interaction between PDAC cells and macrophages, a trans-well co-culture setup was used. The macrophages were seeded on permeable trans-wells at a cell count of 3 × 10^5^ cells per well. Panc 10.05 and SW 1990 PDAC cells were seeded on separate 6-well plates at a cell count of 5 × 10^5^ cells per well. After 48 h, trans-wells containing macrophages were transferred to 6-well plates containing seeded Panc 10.05 or SW 1990 PDAC cells. Eight treatment/control groups were included for each PDAC cell line as described in Table 1. The serum-free media (SFM) with the respective treatment was then added to each well, with the volume being split evenly, i.e., 1 mL of treatment media to each trans-well. This set-up was then incubated at 37°C, 5% CO_2_.

**Table 1.** The different treatment/control groups included for each designated co-culture setup. All treatments were conducted for 24 h.

Group	Description of PDAC treatment
L	Stimulated with 1 μg/mL LPS
A	Treated with 5 μM ATP
M	Treated with 10 μM MCC950
LA	Stimulated with 1 μg/mL LPS in the presence of 5 μM ATP
LM	Stimulated with 1 μg/mL LPS in the presence of 10 μM MCC950
AM	Stimulated with 5 μM ATP in the presence of 10 μM MCC950
LAM	Stimulated with 1 μg/mL LPS in the presence of 5 μM ATP and 10 μM MCC950
C	RPMI SFM only (control)

### Cell viability assay

After treatment for 24 h, the effect of various treatments (Table 1) on PDAC cell proliferation was examined using MTT Reagent (Merck Calbiochem®, Germany). The treatment media were aspirated, and 1 mL of MTT reagent was added to each well containing PDAC cells. This was incubated at 37°C, 5% CO_2_ for 4 h. The reagent was carefully aspirated, leaving the formed crystals in the well. Following solubilization of the crystals using DMSO, the absorbance was measured at 670 nm using a microplate reader (Tecan Infinite 200 PRO, Switzerland).

### ELISA and Western blot analysis

The cell-free supernatants were collected from each treatment/control group. The levels of IL-1β and TNF-α in the human PDAC cells and mouse macrophages were determined using human (Cat no: RAB0273) and mouse (Cat no: RAB0308) ELISA Kits (Sigma-Aldrich, USA), respectively, according to the manufacturer’s protocol. The determined concentrations were then standardized with the total protein concentration of each group determined using Bradford Assays (Bio-Rad Laboratories Inc., USA).

Following the detachment of cells from their respective wells, PDAC cells and macrophages were lysed using a cell extraction buffer. The protein concentration was determined using the Bradford Assay. Proteins (30 µg) from each sample were subjected to sodium dodecyl sulfate-polyacrylamide gel electrophoresis (SDS-PAGE) at 10% bis-acrylamide resolving gel concentration. The separated proteins were then blotted onto a polyvinylidene difluoride membrane at 100 V for 1.5 h. Transferred membranes were blocked with Blocking One solution (Nacalai Tesque, Japan) for 1 h and incubated with the primary NLRP3 (Cat no: HPA012878) and β-actin (Cat no: SAB5600204) (as endogenous control) rabbit monoclonal antibodies (1:1000 dilution; Cell Signaling Technology, USA) overnight at 4°C under gentle shaking. The membranes were then washed with Tris Buffered Saline-Tween (TBS-T) three times over 10-minute intervals and incubated with a secondary, HRP-linked antibody (1:2000 dilution; Cell Signaling Technology, USA) for 1 h at room temperature under gentle shaking. Subsequently, the membranes were incubated with Chemi-Lumi One Super ECL reagent (Nacalai Tesque, Japan) and visualized for densitometric analysis using the Bio-Rad Calbiochem® XRS+ system and Bio-Rad Image Lab 6.1 software. All Western blots were performed in triplicate.

### Statistical analysis

GraphPad Prism 8.0 statistical software was used to analyze the data. The statistical significance of difference in the means was assessed using the one-way analysis of variance (ANOVA) and Tukey’s post hoc test. Differences with p-values less than 0.05 were considered to be statistically significant.

## Results

### Effects of MCC950 on cell proliferation of LPS-stimulated PDAC cells co-cultured with macrophages

**Figure 1.**
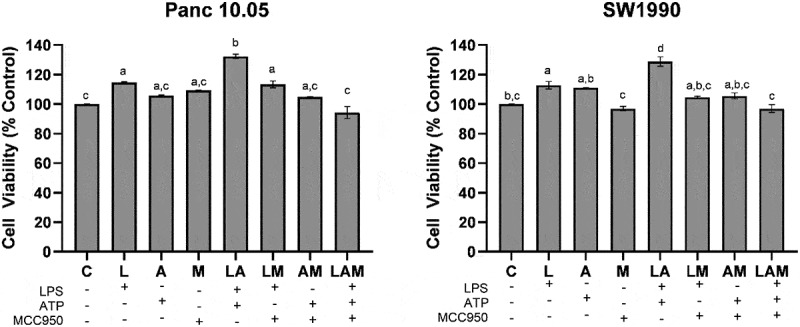
Cell proliferation of Panc 10.05 and SW 1990 cells co-cultured with macrophages under LPS-stimulated inflammation with or without ATP and MCC950.

In PDAC cells/macrophages co-cultures, there was a significant (p < .05) increase in the proliferation of the PDAC cells under 1 μg/mL LPS stimulation (L) when compared with the control (C) (Figure 1). The increase in cell proliferation was significantly greater upon the stimulation by LPS in the presence of 5 μM ATP (LA group). However, when MCC950 was included in addition to LPS and ATP (LAM), no significant difference (p > .05) was observed in PDAC proliferation when compared to control. Interestingly, no significant change in SW 1990 cell proliferation was observed with LPS stimulation in the presence of MCC950 (LM) although an increase in Panc10.05 cell proliferation was noted when compared to control (Figure 1).

PDAC cell lines were co-cultured with macrophages and the proliferation of the PDAC cells was determined by performing the MTT Assay. Data are presented as mean ± SEM of three individual experiments. Means with the same letter(s) above the bar are not significantly different from each other (*p*>.05), whereas means with different letters are significantly different from each other.

### Effects of MCC950 on IL-1β and TNF-α production by LPS-stimulated PDAC cells co-cultured with macrophages

**Figure 2.**
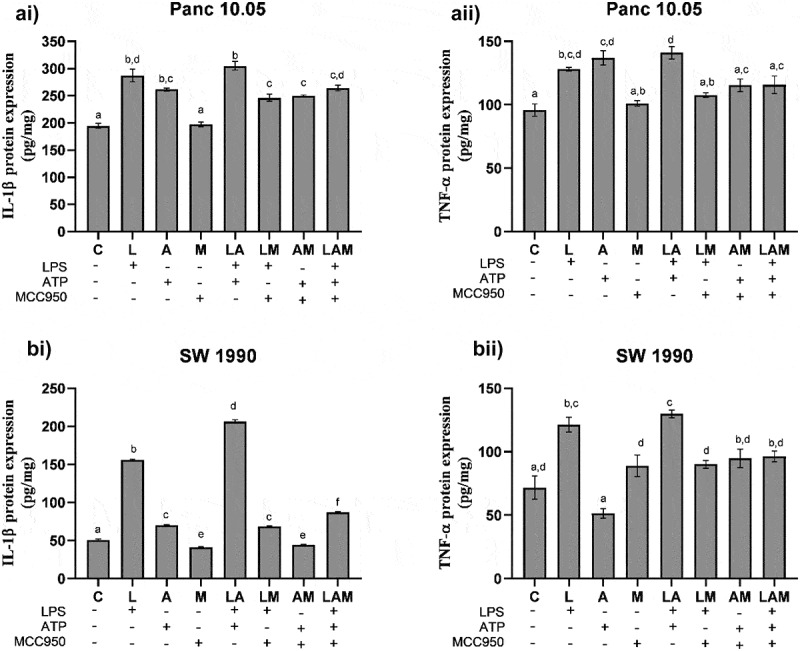
Levels of cytokine a) IL-1β and b) TNF-α production by i) Panc 10.05 PDAC cells and ii) SW 1990 PDAC cells co-cultured with macrophages under LPS-stimulated inflammation, with or without ATP and MCC950.

Compared with the control, a significant increase in the levels of human IL-1β and TNF-α (*p* < .05) was detected in the supernatants obtained from L, A, and LA groups (Figure 2) for both SW 1990 and Panc 10.05 cells co-cultured with macrophages. Interestingly, the addition of MCC950 to these treatments (LM, AM, and LAM) resulted in no significant changes in the levels of TNF-α when compared with control (C). Although addition of MCC950 could reduce the levels of IL-1β in LM, AM, and LAM when compared with L, A, and LA, respectively, it was still significantly higher than the control (C). Furthermore, a significant increase in IL-1β production by SW 1990 cells was noted in the two-step activation by LPS and ATP (LA) compared to single treatment in L and A groups.

Data are presented as mean ± SEM of three individual experiments. Means with the same letter(s) above the bar are not significantly different from each other (*p*>.05), whereas means with different letters are significantly different from each other.

### IL-1β and TNF-α production by macrophages in co-cultured PDAC cells under LPS-stimulated inflammation

**Figure 3.**
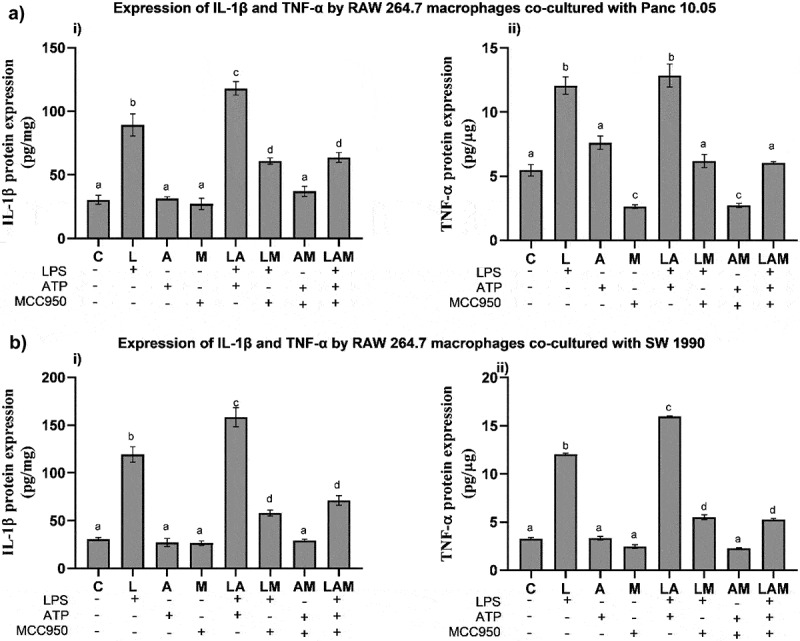
Levels of cytokines i) IL-1β and ii) TNF-α production by RAW 264.7 macrophages in the co-cultures A) Panc 10.05 PDAC cells/RAW 264.7 macrophages and B) SW 1990 PDAC cells/RAW 264.7 macrophages, under LPS- and ATP-induced inflammation with or without NLRP3 inhibition by MCC950.

In PDAC cells/macrophages co-cultures, the levels of mouse IL-1β and TNF-α in the cell supernatants were significantly increased in L and LA groups when compared to the control (C) (Figure 3). The addition of MCC950 significantly reduced the levels of IL-1β and TNF-α in LM and LAM when compared, respectively, with L and LA. However, the cytokine levels recorded in LM and LAM were significantly higher than in control except for TNF-α levels detected in macrophages co-cultured with Panc 10.05 (Figure 3Aii) where no significant differences were noted from the control.

Data are presented as mean ± SEM of three individual experiments. Means with the same letter(s) above the bar are not significantly different from each other (*p*>.05), whereas means with different letters are significantly different from each other.

### NLRP3 expression by Panc 10.05 and SW 1990 PDAC cells under LPS-stimulated inflammation

**Figure 4.**
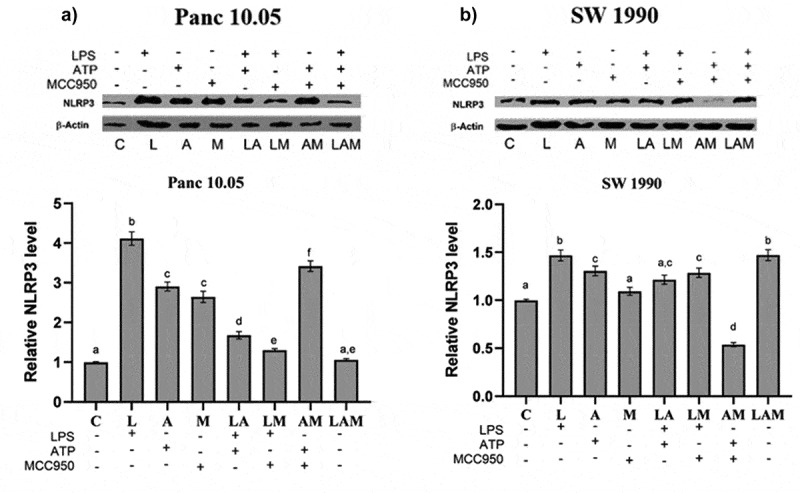
Relative protein expression levels of NLRP3 inflammasome by a) Panc 10.05 and b) SW 1990 PDAC cells in their respective co-cultures with RAW 264.7 macrophages under LPS and ATP-induced inflammation with or without MCC950 inhibition were analysed using Western blot.

A significant increase in the levels of NLRP3 was observed in cell lysates of both PDAC cells when co-cultured with macrophages under LPS-stimulated inflammation or/and ATP treatment (L, A, and LA) although the two-step activation provided by LA did not result in higher production of NLRP3. The addition of MCC950 reduced the levels of NLRP3 in LM and LAM in Panc 10.05 cells when compared, respectively, with L and LA but increased the NLRP3 levels in AM when compared to A (Figure 4a). However, MCC950 reduced the NLRP3 levels in SW 1990 PDAC cells from LM and AM groups when compared to L and A, respectively. Interestingly, MCC950 increased the NLRP3 levels in SW 1990 PDAC cells from the LAM group when compared to LA (Figure 4b).

β-actin expression serves as the control. Data are presented as mean ± SEM of three individual experiments. Means with the same letter(s) above the bar are not significantly different from each other (*p*>.05), whereas means with different letters are significantly different from each other.

### NLRP3 expression by macrophages in co-culture with PDAC cells under LPS-stimulated inflammation

**Figure 5.**
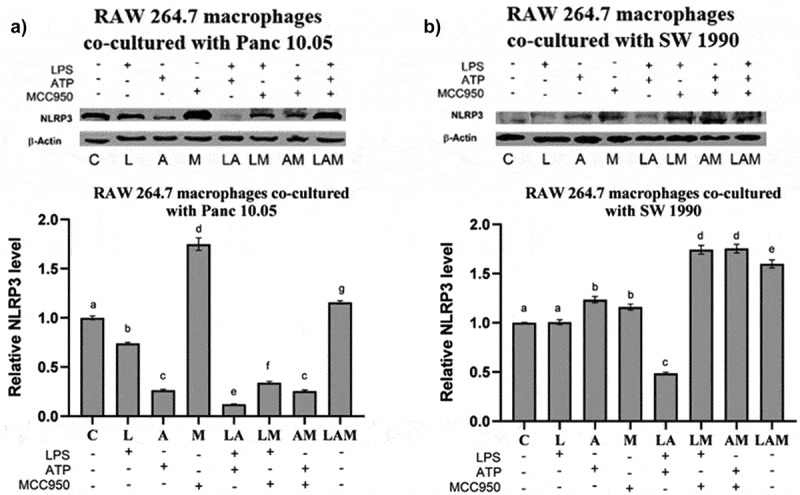
Relative levels of NLRP3 inflammasome by macrophages when co-cultured with a) Panc 10.05 cells and b) SW 1990 cells under LPS-stimulated inflammation with or without ATP and MCC950. Proteins in cell lysates of each treatment were subjected to the Western blot analysis.

In Panc 10.05 cells/macrophages co-cultures, there was a significant decrease in the levels of NLRP3 in macrophages in all treatment groups when compared with the control, except for M and LAM (Figure 5a). LPS stimulation or ATP treatment with or without MCC950 caused a reduction in the expression of NLRP3 in macrophages, but MCC950 treatment alone (M) or combined with both LPS and ATP (LAM) significantly increased NLRP3 levels in macrophages when compared with control.

Levels of NLRP3 were normalized with the levels of β-actin, which served as the endogenous control. Data presented as mean ± SEM of three individual experiments. Means with the same letter(s) above the bar are not significantly different from each other (*p*>.05), whereas means with different letters are significantly different from each other.

On the contrary, in SW 1990 cells/macrophage co-cultures, a significant increase in the levels of NLRP3 in macrophages was observed in all treatment groups when compared with control, except L and LA (Figure 5b). In fact, the presence of LPS and ATP (LA) significantly reduced the NLRP3 levels when compared to control. Moreover, the addition of MCC950 in the treatment with LPS or/and ATP (LM, AM, and LAM) significantly increased the levels of NLRP3 in macrophages co-cultured with SW 1990 cells when compared with their respective counterparts (L, A, and LA).

## Discussion

Chronic inflammation has long been considered a risk factor in cancer development and progression.^13,42^ In the pancreas, chronic inflammation is known to promote an inflammatory milieu that can destabilize the acinar cell genome, hence encouraging the accumulation of oncogenic mutations.^43^ The central mechanism driving this inflammation in immune cells is proposed to be orchestrated by the inflammasome.^44,45^ However, to date, it remains unresolved how the interaction between PDAC and macrophages regulates the NLRP3 inflammasome and pro-inflammatory cytokines in the tumor microenvironment and ultimately its effect on PDAC progression.

**Figure 6.**
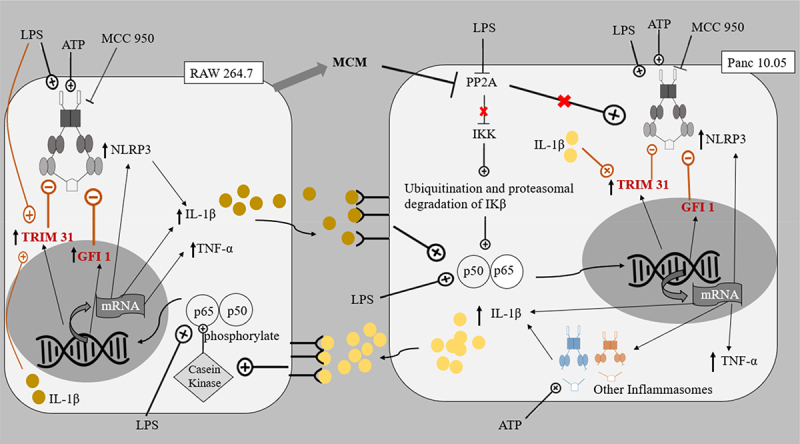
The proposed pathways of interaction between Panc 10.05 PDAC cells and RAW 264.7 macrophages.

**Figure 7.**
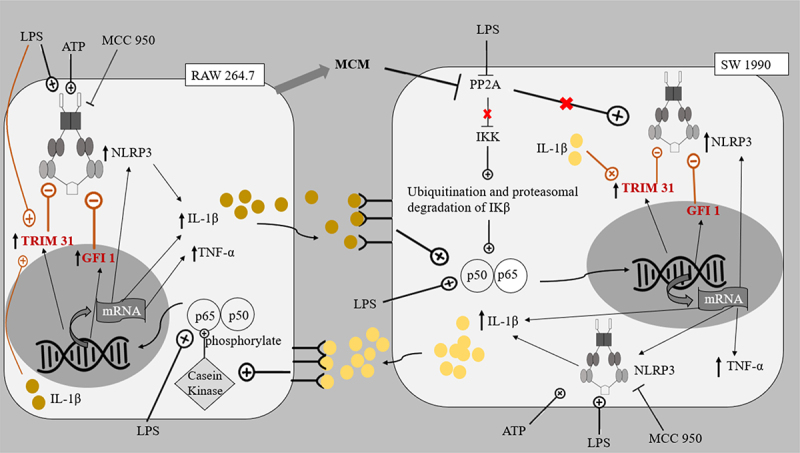
The proposed pathways of interaction between SW 1990 PDAC cells and RAW 264.7 macrophages.

In this study, elevated levels of IL-1β (about 200–300 pg/mg) and TNF-α (about 100–150 pg/mg) were produced by PDAC cells and macrophages under co-culture conditions in the presence of LPS (L and LA) (Figures 2, 3). The increase in cytokines was in accordance with the reported ability of LPS to activate NF-κB in all cell lines, leading to downstream transcription of the cytokines and inflammasomes.^21^ Although an increased level of cytokines was also reported in the monoculture of LPS-induced PDAC cells, the level of IL-1β was only approximately 6 pg/mg.^46^ This elevated production level of IL-1β observed in PDAC cells/macrophages co-cultures suggested that the regulation of the NLRP3 inflammasome between macrophages and PDAC cells could positively regulate cytokines, particularly IL-1β production, contributing to the inflammatory microenvironment. Studies have shown that the stimulation of macrophage IL-1 receptors by IL-1β produced by PDAC cells could lead to the activation of Casein Kinase I and II in macrophages, which in turn phosphorylated the p65 subunit of NF-κB heterodimer, resulting in the increased activation of NF- κB.^47^ The activated NF-κB might then trans-locate into the nucleus and stimulate the transcription of the mRNA of inflammatory components, such as pro-IL-1β, TNF-α, the NLRP3 inflammasome, and other inflammasomes (Figures 6, 7).^48^

This system emulates the interaction between primary adenocarcinoma cells (Panc 10.05) and TAMs in an isolated microenvironment in the absence of other immune cells and stromal cells. Central to the co-culture model would be the role of macrophage-conditioned medium (MCM) in inhibiting PP2A repression, leading to increased activity of kinases, including IKK, JNK, PKC, and ERK. The roles of LPS, ATP, MCC950, TRIM 31, and GFI1 on the negative regulation of NLRP3 inflammasome were also appreciated. MCM: Macrophage Conditioned Medium; PP2A: Protein Phosphatase 2A; IKK: IκB kinase; JNK: c-Jun N-terminal Kinases; PKC: Protein Kinase C; ERK: Extracellular signal Related Kinase; LPS: Lipopolysaccharide; ATP: Adenosine Triphosphate; TRIM31: Tripartite Motif Containing 31; GFI1: Growth Factor Independence 1; p50-p65: NF-κB.

This system emulates the interaction between primary adenocarcinoma cells (SW 1990) and TAMs in an isolated microenvironment in the absence of other immune cells and stromal cells. Central to the co-culture model would be the role of macrophage-conditioned medium (MCM) in inhibiting PP2A repression, leading to increased activity of kinases, including IKK, JNK, PKC, and ERK. The roles of LPS, ATP, MCC950, TRIM 31, and GFI1 on the negative regulation of NLRP3 inflammasome were also appreciated. MCM: macrophage conditioned medium; PP2A: protein phosphatase 2A; IKK: IκB kinase; JNK: c-Jun N-terminal kinases; PKC: protein kinase C; ERK: extracellular signal-related kinase; LPS: lipopolysaccharide; ATP: adenosine triphosphate; TRIM31: tripartite motif containing 31; GFI1: growth factor independence 1; p50-p65: NF-κB.

The expressed cytokines could further contribute to the tumor microenvironment between macrophages and PDAC cells by repressing PP2A in PDAC cells.^49,50^ PP2A protein dephosphorylates and inactivates the IKK complex keeping NF-κB activation in check, hence the repression of PP2A leads to uninhibited activation of NF-κB in PDAC cells. As mentioned in previous reports, the reduced inhibition of NF-κB activation meant that along with the existing KRAS and STAT3 cytokine networks, a greater sustained activation of NF-κB ensues.^51^ PP2A repression also increases the activity of kinases including IKK, JNK, PKC, and ERK, all of which have important roles in accelerating pancreatic cancer progression.^50,52–54^ In accordance with these reports, this study showed that the cell proliferation of PDAC cells increased when treated with LPS and/or ATP (Figures 1, Figures 5, Figures 6, 7).

Following MCC950 treatment (M), the cytokine production by PDAC cells and macrophages was significantly reduced. This could be due to the role of MCC950 as a highly specific inhibitor of ATP hydrolysis by the NLRP3 inflammasome.^55^ The inhibition of ATP hydrolysis, prevents or reverses conformational changes in NLRP3 protein necessary for inflammasome assembly and activation. The addition of LPS, however, (LM) offsets the inhibition by MCC950 in the production of IL-1β by PDAC cells (Figure 3Bii). This could be due to the increase in NF-κB activation in the macrophages and hence greater transcription and activation of NLRP3 inflammasome in both cell lines.^56^ ATP on its own did not massively increase the production of IL-1β in SW 1990 PDAC cells and RAW 264.7 macrophages as the two-step activation is required for NLRP3 inflammasome (Figures 2, 3). Unlike the SW 1990 metastatic cancer cell line, in Panc 10.05, induction with LPS and ATP individually or together (L, A, and LA groups) resulted in a similar increase in the production of IL-1β (Figure 2). This is in accordance with previous studies, as primary PDAC cells (Panc 10.05) lack ASC meaning, the NLRP3 inflammasome is not largely responsible for the production of IL-1β in this cell line and instead other inflammasomes play a prominent role in the cleavage of pro-IL-1β into IL-1β.^46^ The production of TNF-α by the PDAC cells was significantly higher in co-cultures stimulated by LPS, ATP, and LA for Panc 10.05 and LPS and LA for SW 1990 PDAC cells, when compared to control. Otherwise, no significant change in the production of TNF-α was observed. This observation highlights the role of TNF-α in sustaining a pro-inflammatory microenvironment and its synergism with IL-1β.^38^ It was also noted that the production of both IL-1β and TNF-α, by RAW 264.7 macrophages, showed a similar trend in the different treatment groups, relative to control.

In addition, in this study, LPS-induced Panc 10.05 PDAC cells (L, LA, and LM groups) demonstrated an increase in proliferation compared to their control counterparts (Figure 1). This confirms the ability of LPS to further repress PP2A expression and promote IL-1β production, effectively stimulating NF-κB activation, which in turn enhances mitogenic EGF receptor signaling.^5,10^ This also correlated with the significant increase in the expression of NLRP3 in Panc 10.05 PDAC cells, following treatment with LPS and/or ATP (L, A, LA, and LM groups) (Figure 4). The level of pro-inflammatory cytokines produced by Panc 10.05 PDAC cells and macrophages was also increased in the LPS-stimulated co-cultures (L, LA, and LM groups), suggesting that the increased levels of pro-inflammatory cytokine in the tumor microenvironment also contributed to the above-mentioned PP2A repression mechanism, resulting in an increased cell proliferation. Treatment with MCC950 did not prevent the increase in pro-inflammatory cytokines in the tumor microenvironment, nor the increase in proliferation of Panc 10.05 PDAC cells in LPS-stimulated pro-inflammatory microenvironment (Figure 6).

Similarly, LPS-induced SW 1990 PDAC cells (L and LA groups) demonstrated a similar increase in cell proliferation. However, the addition of MCC950 to inflammatory stimuli LPS (LM and LAM groups) reduced the level of IL-1β in the tumor microenvironment by the SW 1990 PDAC cells and macrophages, and this could prevent the increase in NF-κB activation in the PDAC cells brought about by the pro-inflammatory microenvironment. Hence, despite the increase in NLRP3 expression in both SW 1990 PDAC cells and macrophages, in these groups, there was no increase in the proliferation of SW 1990 PDAC cells in the presence of MCC950 (Figures 1, Figures 2, 7).

Interestingly, it was observed that NLRP3 levels were generally increased in PDAC cells when treated with LPS and/or ATP (Figure 4) but not in macrophages (Figure 5). Also, in the SW 1990 PDAC cells, there was no significant change in the expression of NLRP3 in the LA group (Figure 4b), suggesting that the massive increase in pro-inflammatory cytokine IL-1β could significantly induce Tripartite Motif Containing 31 (TRIM31) and Growth Factor Independence 1 (GFI1) negative regulation, offsetting the initial increase in transcription. Studies have reported that IL-1β stimulates the mRNA transcription and protein expression of TRIM31, and the downstream-sustained NF-κB activation also leads to the transcription of GFI1. TRIM31, which is also constitutively expressed in macrophages and PDACE cells, promotes the proteasomal degradation of the NLRP3 inflammasome, and GFI1 represses the transcription of NLRP3 inflammasome.^57,58^ The results suggest that for a particular cell, IL-1β present in the microenvironment can stimulate both TRIM31 and GFI-negative regulation. This action of IL-1β is reminiscent of how selective phosphorylation of NF-κB is able to finely tune the inflammatory genome transcription, as phosphorylation of serine-316 residue yields pro-inflammatory cytokines and pro-tumorigenic chemokines such as TNF-α, IL-8, VEGF, and IL-10.^59^ The effects of LPS and ATP on the regulation of NLRP3 expression in PDAC cells in co-cultures were not like those observed in monocultures of the PDAC cells in a study by Yaw et al. This could be due to the additional input from the interaction between the PDAC cells and the macrophages, setting the inflammatory microenvironment.

Our observations led to the understanding that the levels of NLRP3 expression in cells are finely controlled by the PDAC cells and macrophages via the same regulators (e.g., IL-1β), with different effects at different concentrations. The differing effects of MCC950 in decreasing the expression of NLRP3 in the LA group of primary Panc 10.05 PDAC cells and in increasing the expression of NLRP3 in the LA group of metastatic SW 1990 PDAC cells depict that the role of MCC950 in the co-culture model depends on whether the particular PDAC cell line relies on the NLRP3 inflammasome for background IL-1β production. The lesser the PDAC cell line relies on NLRP3 inflammasome for background IL-1β production, the greater the level of inflammatory cytokines in the tumor microenvironment and the greater the extent of negative feedback on NLRP3 expression, which could not be offset by MCC950 inhibition on cytokine production.

In both co-cultures, there was a significant decrease in the expression of NLRP3 inflammasome in RAW 264.7 macrophages in the background of increased levels of pro-inflammatory cytokines in the tumor microenvironments stimulated by LPS and ATP (Figure 5a, b). This highlights the prominent role of the negative regulators, e.g., TRIM31 and GFI1, and the effect of the microenvironment on these negative regulators.^48,57^ Despite the initial induction of NF-κB to stimulate transcription of NLRP3 inflammasome, the massive production of cytokines by both cell lines could increase the levels of TRIM31 and GFI1, which in turn decreased the expression of NLRP3. Our results also suggest that the negative regulation of NLRP3 inflammasome is relatively greater in RAW 264.7 macrophages compared to PDAC cells.

In Panc 10.05 cells/macrophages co-cultures, the addition of LPS or ATP to MCC950 (AM and LM) significantly reduced the NLRP3 inflammasome expression in the macrophages. On the contrary, in SW 1990 cells/macrophages co-cultures, a significant increase in NLRP3 expression in macrophages was observed in the AM and LM groups. This variation between the co-cultures could be due to Panc 10.05 exerting greater negative feedback, as it produces more IL-1β than the MCC950-inhibited SW 1990, leading to possibly increased TRIM31 induction in the macrophages. This highlights the impact of NLRP3 inflammasome activity on the interaction between PDAC cells and macrophages and their tumor microenvironment.

However, in order to understand the tumor microenvironment better with the ultimate aim of introducing a new, safe, and efficacious clinical chemotherapeutic drug, the regulation of PDAC tumor cells microenvironment by the NLRP3 inflammasome needs to be further studied *in vivo* and in human cells.

## Conclusion

In the co-culture treatments, pro-inflammatory cytokines such as IL-1β modulate the interaction between the PDAC cells and macrophages under LPS stimulation, as highlighted in this study. The NLRP3 expression in both PDAC cell lines and macrophages, under LPS-stimulated inflammation, was regulated by the microenvironment. The interaction between the PDAC cells and macrophages resulted in a pro-inflammatory environment with elevated levels of IL-1β and TNF-α, as well as increased the proliferation of PDAC cells. The proliferation of PDAC cells was closely related to the level of pro-inflammatory cytokines in the tumor microenvironment, rather than the level of NLRP3 expression in PDAC cells and macrophages. The addition of MCC950 did not reduce the effects of LPS or/and ATP treatment on primary Panc 10.05 cell proliferation although the cytokine levels were reduced. However, in the presence of MCC950, metastatic SW 1990 PDAC cell proliferation was significantly reduced in the presence of LPS and ATP. Hence, the potential therapeutic efficacy of MCC950 in treating metastatic PDAC cells relying on the NLRP3 inflammasome to regulate their microenvironment and interaction with macrophages must be studied further.

## Abbreviations

A/ATP: Adenosine Triphosphate
ADM: Acinar-to-Ductal Metaplasia
AM: Combination of Adenosine Triphosphate and MCC950
ASC: Apoptosis-associated Speck-like protein containing a CARD complex
C2TA: MHC Class 2 Transcription Activator
CARD: Caspase Activation and Recruitment Domain
CCL2: Chemokine (C-C motif) Ligand 2 (CCL2)
CCR2: C-C Chemokine Receptor type 2
CSC: Cancer Stem Cells
DAMP: Damage Associated Molecular Pattern
ECM: Extracellular Matrix
ERK: Extracellular signal Related Kinase
FADD: FAS-mediated death domain protein
GFI1: Growth Factor Independence 1
HET: Heterokaryon Incompatibility Gene het
iCAF: Inflammatory Cancer Associated Fibroblasts
IKK: IκB-kinase
IL: Interleukin
IP-10: Interferon γ-induced Protein 10
JNK: c-Jun N-terminal Kinases
KRAS: Kirsten Rat Sarcoma viral oncogene
L/LPS: Lipopolysaccharide
LAM: Combination of Lipopolysaccharide, Adenosine Triphosphate and MCC950
LM: Combination of Lipopolysaccharide and MCC950
LRR: Leucine Rich Repeat
M: MCC950
mCAF: Myofibroblastic Cancer Associated Fibroblasts
MCM: Macrophage Conditioned Medium
mtDNA: Mitochondrial DNA
NAIP: Neuronal Apoptosis Inhibitor Protein
NEK: NIMA Related Kinase
NF-κB: Nuclear Factor kappa B
NLR: NOD Like Receptor
NLRP3: NOD Like Receptor family, Pyrin domain – containing 3
NOD: Nucleotide binding Oligomerization Domain; Also known as NACHT
PAMP: Pathogen Associated Molecular Pattern
PanIN: Pancreatic Intraepithelial Neoplasia
PDAC: Pancreatic Ductal Adenocarcinoma
PDGF: Platelet Derived Growth Factor
PKC: Protein Kinase C
PP2A: Protein Phosphatase 2A
PRR: Pattern Recognition Receptors
ROS: Reactive Oxygen Species
TAM: Tumour Associated Macrophages
TGF-β: Transforming Growth Factor β
TLR: Toll-Like Receptor
TNF: Tumour Necrosis Factor
TP1: Telomerase-associated Protein
TRIM31: Tripartite Motif Containing 31
VEGF: Vascular Endothelial Growth Factor
